# Supplementary material for: A machine learning model of microscopic agglutination test for diagnosis of leptospirosis
Source: PLoS One. 2021 Nov 16;16(11):e0259907. doi: 10.1371/journal.pone.0259907 (PMC8594833; doi:10.1371/journal.pone.0259907)
Supplement: S1 File — (DOCX) [file pone.0259907.s001.docx]

# Supporting information

## Binary classification

Binary classification categorizes each data either as positive or negative and consists of training and test steps. S1 Fig shows an example of 2-dimensional non-linearly separable and inseparable data. S1A shows a good data where positive and negative data are separably distributed. The training step makes several potential boundaries (curves in S1B) and selects the boundary that separates the training data with the highest classification score (the green curve in S1B). As shown in the figure, the selected boundary perfectly separates the negative and positive data. The test step evaluates the selected boundary with test data (S1C). As shown in the figure, the selected boundary both negative and positive data are perfectly separated, which means the trained classifier is good. The success of binary classification heavily depends on the distribution of data. S1D-S1F show how badly distributed data worsen the performance of binary classification. Contrast to the good case, S1D shows bad data where positive and negative data are inseparably distributed. None of the potential boundaries cannot perfectly separate even the training data (S1E and S1F).

Pre-processing on MAT images

We applied the following pre-processing to all the MAT images that have different properties as shown in Table S1. To apply the pre-processing, all the extracted patches are under similar lighting conditions and the same meter-resolution scale.

We have first applied scale normalization in order to make all the images in the dataset which has the same micrometer-resolution scale. The original dataset is made of a series of negative images captured under 20× objective lens (50 micrometer scale) and of positive images with 40× objective lens (20 micrometer scale) (Fig. S2A and B). They contain the same resolution; however, the pixels applied to 50 micrometers in the negative images are 290, whereas to 20 micrometers in the positive images are 232. Based on the micrometer-resolution scale indicator shown in each image, this preprocessing resizes them to have the same micrometer-pixel scale (Fig. S2C and D). The number and resolutions of images in each dataset were shown in Table S2.

Each scale-normalized image was cut into smaller patches (256 × 256 or 512 × 512). Each patch must be large enough to count the amount of agglutination and must avoid too large resolution to fulfill the minimum requirement of computers.

Finally, data standardization was applied to each patch to satisfy an assumption that all feature vectors are centered around zero and have similar variance. For instance, if the data acquisition process affects the brightness of observed pictures, then the standardization is applied to cancel those unwilling effects. Suppose we have a feature vector $x\in R^{d}$ representing an image patch. Data standardization normalizes the feature vector such that the normalized vector has zero mean and unit variance as $\hat{x}=\frac{\{x-\mu_{x}\}}{\sigma_{x}}$, where $\mu_{x}$and $\sigma_{x}$ denote the mean and standard deviation of a set of $x$.

### K-fold cross validation

K-fold cross validation is a well-used practical solution to avoid overfitting that a trained classifier performs well against training data but much worse against unseen test data. Figure S3 shows the concept of K-fold cross validation. All data is split into either training or test datasets. The training dataset is further divided into $K$ group, each of which is called a fold. For each fold, an SVM is trained with the remaining $K-1$ folds and is validated with the fold. The validation errors of all folds are averaged to measure generalization performance of the model while cancelling overfitting for specific data. After the training, the trained SVM is further validated with the test dataset.
