# Supplementary figures and images for: A machine learning model of microscopic agglutination test for diagnosis of leptospirosis
Source: PLoS One. 2021 Nov 16;16(11):e0259907. doi: 10.1371/journal.pone.0259907 (PMC8594833; doi:10.1371/journal.pone.0259907)

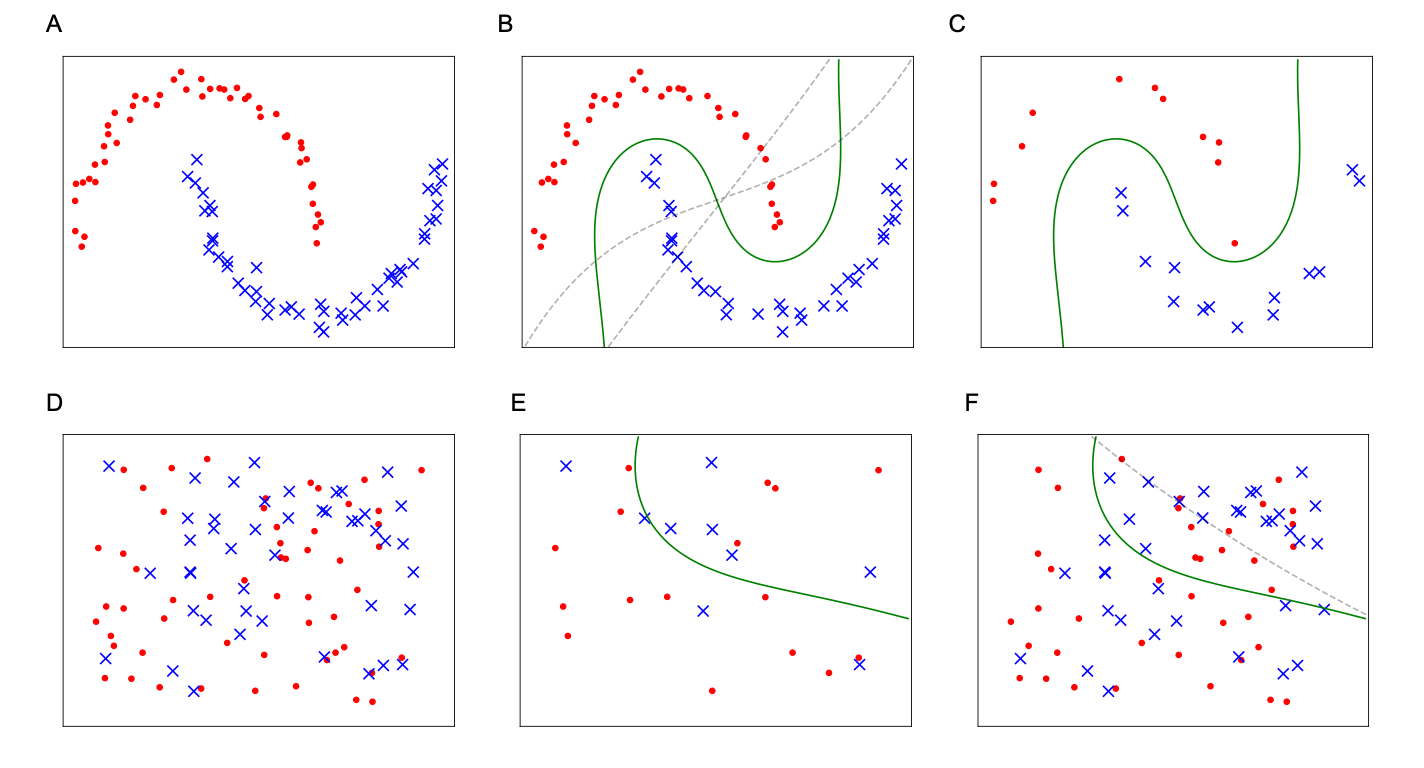

Supplement: S1 Fig — Red circle and blue cross symbols represent negative and positive data. Curved lines represent boundaries obtained from training data of which green lines represent the best boundary. (A) A good training data. (B) Potential boundaries obtained from (A). (C) The best boundary obtained from (A). (D) A bad training data. (E) Potential boundaries obtained from (B). (F) The best boundary obtained from (B). (TIF) [file pone.0259907.s002.tif]

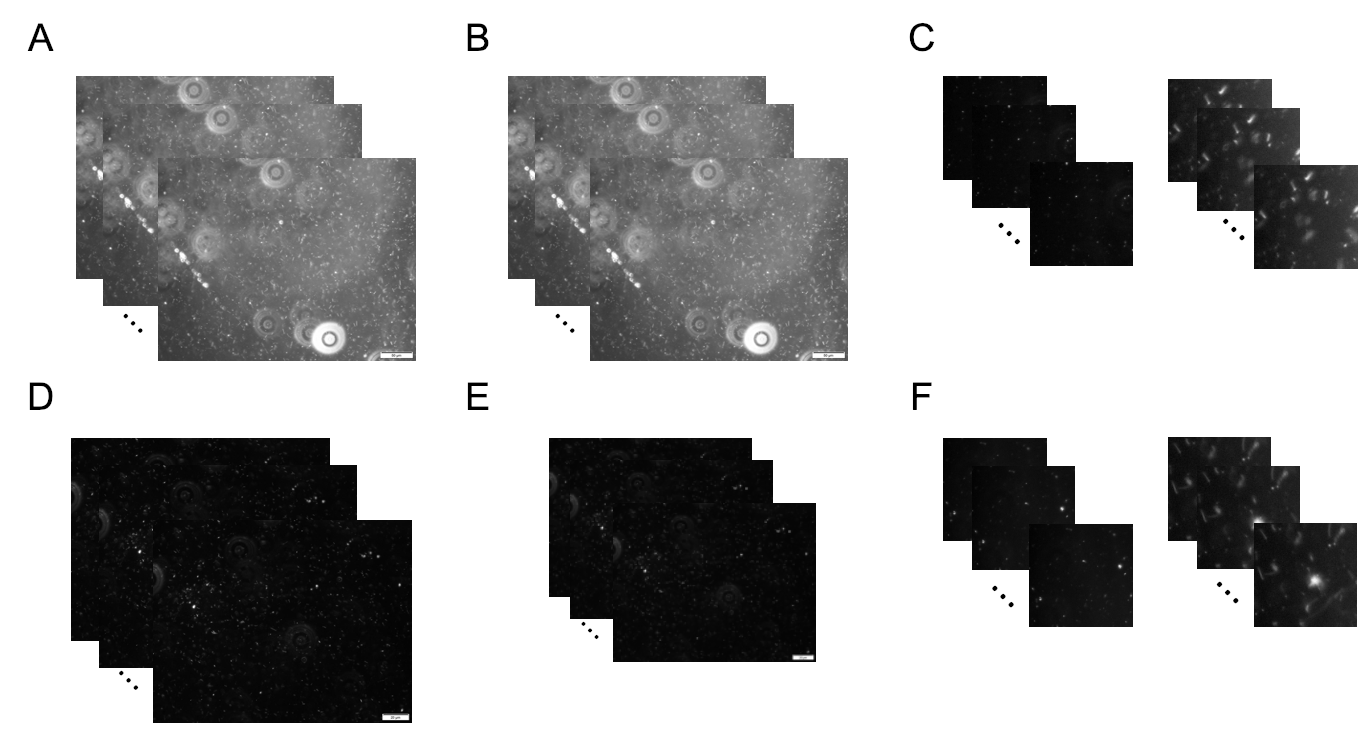

Supplement: S2 Fig — (A) Raw MAT images of Negative data. (B) Scale normalized images of Negative data. (C) Extracted patches of Negative data. (D) Raw MAT images of Positive data. (E) Scale normalized images of Positive data. (F) Extracted patches of Positive data. (TIF) [file pone.0259907.s003.tif]

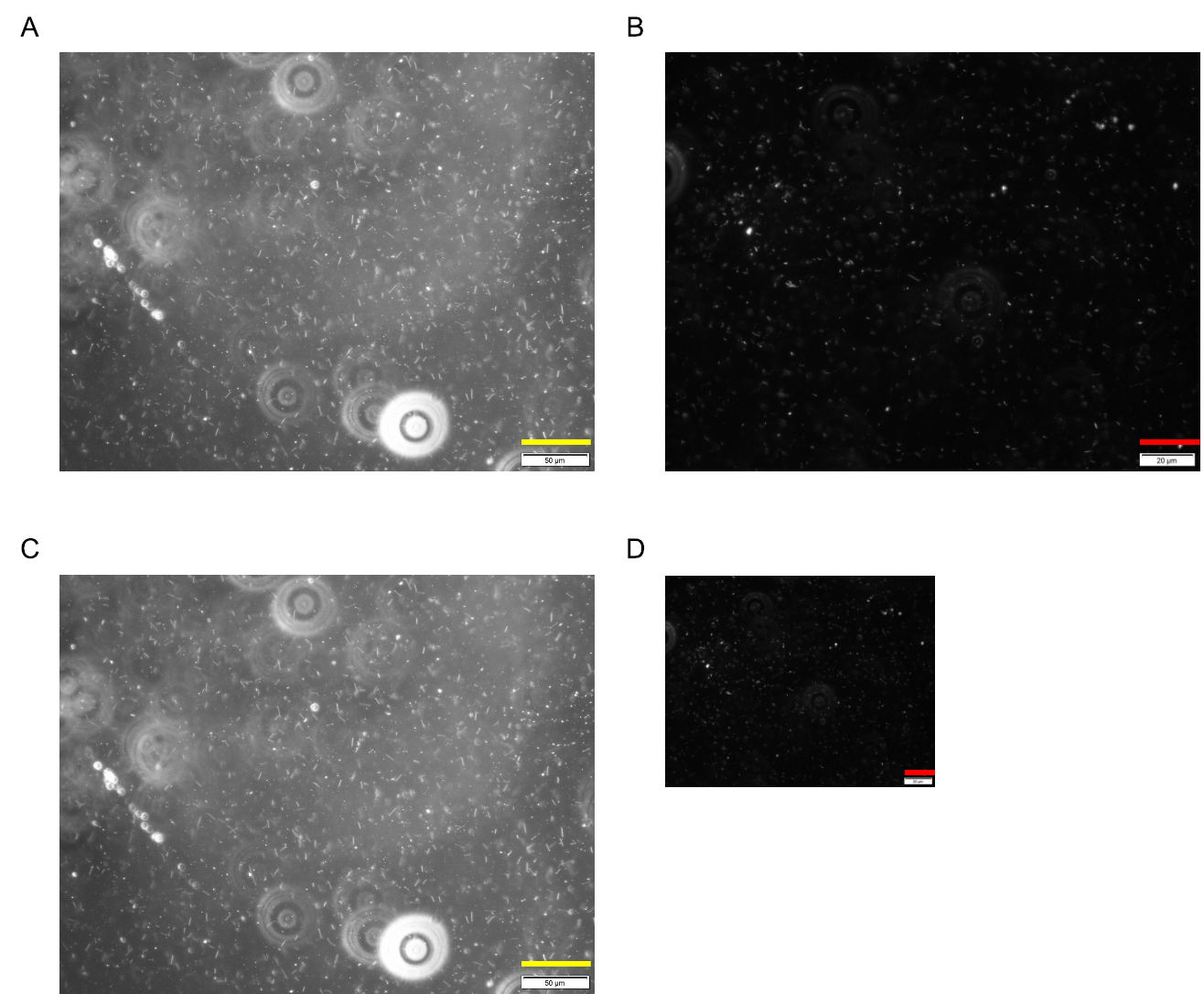

Supplement: S3 Fig — Yellow and red scale bars represent 50 and 20 micrometers respectively. (A) A raw MAT image of Negative data. (B) Image (A) with scale normalized. (C) A raw MAT image of Positive data. (D) Image (C) with scale normalized. (TIF) [file pone.0259907.s004.tif]

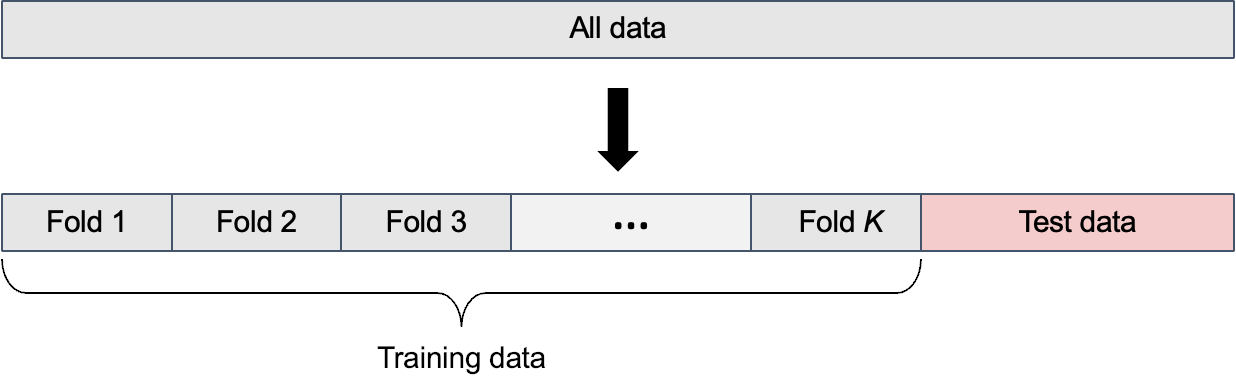

Supplement: S4 Fig — (TIF) [file pone.0259907.s005.tif]

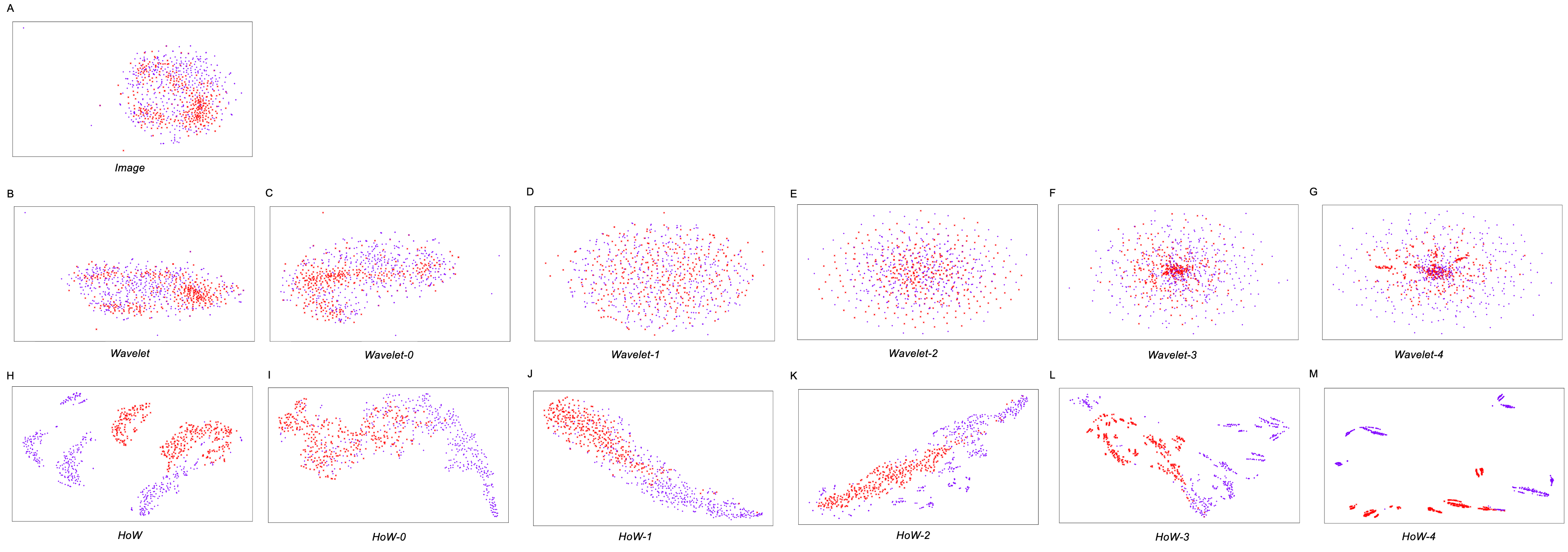

Supplement: S5 Fig — Red (x) and purple (+) symbols represent the features of negative and positive patches, respectively. (TIF) [file pone.0259907.s006.tif]
