# Supplementary material for: A machine learning model of microscopic agglutination test for diagnosis of leptospirosis
Source: PLoS One. 2021 Nov 16;16(11):e0259907. doi: 10.1371/journal.pone.0259907 (PMC8594833; doi:10.1371/journal.pone.0259907)
Supplement: Attachment — Submitted filename: Response_to_reviewers_20210514.docx [file pone.0259907.s007.docx]

Dear Drs. Lin and Bastos, editors of PLOS Neglected Tropical Diseases,

We would like to thank all the editors and reviewers very much for reviewing our manuscript. We would like to submit a revised version of our manuscript entitled “A Machine Learning Model of Microscopic Agglutination Test for Diagnosis of Leptospirosis”, manuscript number PNTD-D-20-02149 with all modifications following reviewers’ advice. Our responses to reviewers’ comments are below.

First, we would like to point out the following: the significance of this study is to show that MAT automation is methodologically feasible. This manuscript does not aim to verify the performance of the machine learning algorithm established in this study in a clinical/diagnostic testing environment.

We appreciate that reviewer #2 understands this point, while we admit that our manuscript was misleading in some parts to show the purpose of this paper.

Here, we have revised the manuscript that may explain well the purpose of this study.

We specifically

- Modified the title from "A Machine Learning Approach Towards Standardizing Microscopic Agglutination Test for Diagnosis of Leptospirosis" to “A Machine Learning Model of Microscopic Agglutination Test for Diagnosis of Leptospirosis” to better fit our purpose.

- Modified the abstract and introduction which is detailed below individual comments.

- Revised the results and conclusion.

We understand the concerns of reviewer #1 that we only tested with one serotype, but the purpose of the current paper is to show feasibility of our machine learning model of MAT as a model that we believe the results are sufficient. We appreciate suggestion of reviewer #1 to perform additional experiments that are needed for future practical use. Those experiments are currently being planned; however, those are out of scope of current study, thus, are not included in this revised manuscript.

**Methods**

> Reviewer #1: No, the last paragraph of the introduction says, the goal of the study is to fully automate the MAT procedure. But the study mainly focused on MAT image classification only.

The study only tested one serovar using serum from hamster. It is too early to justify the sensitivity of the technique. The image classification should be performed with at least all WHO panel and also evaluated with actual clinical samples

Thank you for this comment. Construction of the last paragraph of Introduction was misleading as you pointed out. We modified the paragraph to fit the aim of the current paper. The paragraph now reads “This study proposes a machine learning technique in classifying MAT images towards the fully automated MAT. …….This study will be the first step of our ultimate goal that is to fully automate the MAT procedure. “

Full automation of MAT is the “final” goal of our future project, and the aim of this paper is to show the possibility of machine learning diagnosis of images using a model, which is essential for fully automated MAT in the future.

A large-scale evaluation of our model utilizing WHO panel and clinical samples is currently planned but not included in this paper.

>Reviewer #2: Are the objectives of the study clearly articulated with a clear testable hypothesis stated.

Is the study design appropriate, is the population clearly described, is the sample size sufficient, correct statistical analysis and ethical or regulatory requirements were met.

In this study, they built an automatic identification algorithm of MAT using a machine learning method to determine aggregation within microscopic images.

Thank you very much for understanding our aims and fit methodology.

--------------------

**Results**

> Reviewer #1: Many serogroups should be tested and evaluated with actual clinical samples. The agglutination pattern varies for different serogroup

We agree with the need of evaluating various strains when it comes to practical/clinical use. Therefore, we are planning future experiments utilizing the WHO panel and clinical samples in the coming project. However, we believe that testing one strain to show the proposed method can be applied to MAT as a model system which uses the statistical rate of its occurrence as a histogram of wavelet coefficients.

It requires further experimentation and evaluation as pointed out by reviewer #1, but we believe methodology taken fits the purpose of this paper.

Reviewer #2: The are results clearly and completely presented.

The figures (Tables, Images) of sufficient quality for clarity.

Three evaluations were to conducted validate the effectiveness and efficiency of the proposed method: (1) the elapsed time of the feature extraction process, (2) qualitative evaluation of the MAT image feature classification, and (3) quantitative evaluation of the MAT image feature classification.

Thank you very much for your comment. We are very glad that you agreed to our results presentation.

--------------------

**Conclusions**

Reviewer #1: The study is in very initial stage and need to evaluate the sensitivity with actual clinical samples of different titers and different serovars.

We agree with reviewer #1 that our current study is at a very initial stage and needs further assessment for clinical application. However, the importance of this research is that we have built a model that leads to replacing the evaluation of MAT with machine learning. While it is very important to evaluate it with actual clinical samples, we believe it is the next task, therefore it has not been included in this paper.

Reviewer #2: Public health is relevance addressed.

Microscopic agglutination test (MAT) is considered as the standard test for serological diagnosis of leptospirosis.

However, the procedures involved in MAT, especially judging the results (i.e., whether positive or negative) requires highly-trained personnel that making it difficult to adopt as a general test.

In this study, they primarily attempted to standardize MAT by automating the determination process.

The experimental results showed that the proposed machine learning-based pipeline with the derived image feature well-recognized the agglutination in MAT images, especially combined HoW resulted in the MCC score of 1.0, which means perfect prediction. This indicates that the proposed method could substitute the skilled examiners’ knowledge and experience by using machine learning techniques.

Thank you very much for your comment. We are very grateful that you understand and agree with conclusions we have drawn. It is our hope that the methodology depicted in this paper will put the field of diagnosis of leptospirosis forward in the future.

--------------------

**Summary and General Comments**

Reviewer #1: The study is too preliminary with no clear conclusions

As our purpose of the current paper is to establish a model with an algorithm to determine leptospiral infection-positive and -negative samples, we believe that the data presented are sufficient to achieve the aim of this study. We emphasize that this study is the first step towards MAT automation while there are much more evaluations needed for clinical application.

We are submitting a revised version of this paper in the hope that this explains well to show the potential of MAT automation.

Reviewer #2: The manuscript satisfies the PLOS NTDs criteria for publication: originality; importance and relevance to researchers, practitioners, or policy makers in the field of NTDs.

We appreciate your comments and understanding of our study. We trust that this revised manuscript will be a good fit to PLOS NTDs.
